# A Diagnostic Pitfall of the Reversed Halo Sign: Pulmonary Infarction Mistaken for Pneumonia

**DOI:** 10.1002/ccr3.73140

**Published:** 2026-07-12

**Authors:** Daichi Kuwahara, Kazuhiko Iwasaki

**Affiliations:** ^1^ Department of Internal Medicine Kaga Medical Center Kaga Japan; ^2^ Department of Respiratory Medicine Kanazawa University Kanazawa Japan

**Keywords:** bacterial pneumonia, pulmonary embolism, pulmonary infarction, reversed halo sign

## Abstract

The reversed halo sign may occur in both infection and pulmonary infarction. Even when pneumonia is suspected, pulmonary infarction should remain an important differential diagnosis, especially when a subpleural lesion does not improve with antimicrobial therapy.

## 1 Case

A 78‐year‐old woman presented with fever and anorexia. Chest computed tomography (CT) revealed a well‐demarcated subpleural opacity in the right lower lobe, consisting of central ground‐glass attenuation surrounded by a crescent‐shaped rim of consolidation, consistent with the reversed halo sign (RHS) (Figure 1A–C). Because of fever, elevated inflammatory markers, and detection of *Haemophilus influenzae* from sputum, bacterial pneumonia was initially suspected. However, her general condition and radiological findings did not improve despite appropriate antimicrobial therapy.

**FIGURE 1:**
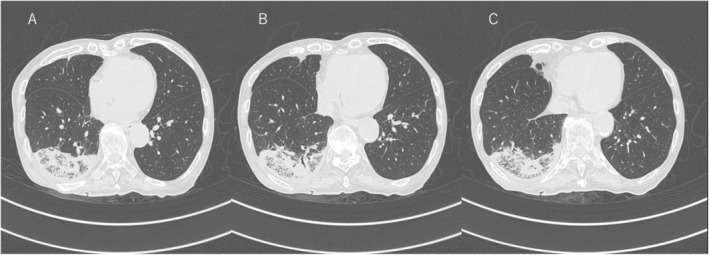
(A–C) Non‐contrast CT shows a subpleural opacity in the right lower lobe with central ground‐glass attenuation surrounded by a crescent‐shaped rim of consolidation, consistent with the reversed halo sign.

Subsequent contrast‐enhanced CT demonstrated a segmental pulmonary arterial filling defect with a corresponding peripheral wedge‐shaped opacity, leading to a diagnosis of pulmonary infarction secondary to pulmonary embolism (Figure 2A–C). Although concomitant infection or airway colonization could not be completely excluded, the clinical course and imaging findings supported pulmonary infarction as the principal cause of the RHS. Anticoagulation therapy with apixaban resulted in gradual radiological improvement, and the patient was discharged.

**FIGURE 2:**
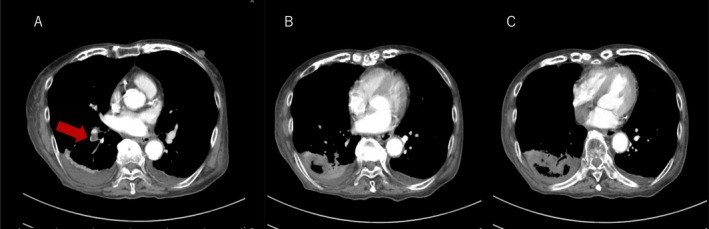
(A–C) Contrast‐enhanced CT demonstrates a segmental pulmonary arterial filling defect with a corresponding peripheral wedge‐shaped opacity, confirming pulmonary infarction. The pulmonary arterial thrombus is indicated by an arrow in (A).

## 2 Discussion

The RHS is a radiological pattern defined by central ground‐glass opacity surrounded by a ring‐shaped or crescentic rim of consolidation. Although it is classically associated with organizing pneumonia, its differential diagnosis is broad and includes infection, granulomatous disease, and thromboembolic disease [1]. In this case, the initial clinical findings and sputum culture supported bacterial pneumonia, and concomitant infection or airway colonization could not be completely excluded. Nevertheless, the absence of improvement after antimicrobial therapy, the demonstration of a pulmonary arterial filling defect, the corresponding wedge‐shaped peripheral opacity, and improvement after anticoagulation indicated pulmonary infarction as the main cause of the RHS.

Pulmonary infarction is an important and potentially underrecognized cause of the RHS. In a retrospective analysis of 286 patients with the RHS, pulmonary infarction was the most frequent etiology, followed by cryptogenic organizing pneumonia and bacterial pneumonia [2]. Therefore, when the RHS is observed, especially as a peripheral subpleural opacity, pulmonary embolism and infarction should be considered even when infection is initially suspected. Contrast‐enhanced CT is useful for avoiding this diagnostic pitfall.

## Author Contributions

**Daichi Kuwahara:** conceptualization, data curation, writing – original draft. **Kazuhiko Iwasaki:** writing – review and editing.

## Funding

The authors have nothing to report.

## Ethics Statement

The authors have nothing to report.

## Consent

Written informed consent was obtained from the patient for publication of this case report and accompanying images.

## Conflicts of Interest

The authors declare no conflicts of interest.

## Data Availability

Data sharing is not applicable to this article as no datasets were generated or analyzed.
